# Comparison of Solid-Phase Extraction Sorbents for Monitoring the In Vivo Intestinal Survival and Digestion of Kappa-Casein-Derived Caseinomacropeptide

**DOI:** 10.3390/foods12020299

**Published:** 2023-01-08

**Authors:** Yunyao Qu, Bum-Jin Kim, Jeewon Koh, David C. Dallas

**Affiliations:** 1 Department of Food Science & Technology, Oregon State University, Corvallis, OR 97331, USA; 2 Nutrition Program, School of Biological and Population Health Sciences, College of Public Health and Human Sciences, Oregon State University, Corvallis, OR 97331, USA

**Keywords:** Kappa-casein-derived caseinomacropeptide, solid-phase extraction, LC-MS/MS, jejunal fluid, whey protein isolate, C18, PGC, HILIC and solid-phase extraction

## Abstract

Kappa-casein-derived caseinomacropeptide (CMP)—a 64-amino-acid peptide—is released from kappa-casein after rennet treatment and is one of the major peptides in whey protein isolate (WPI). CMP has anti-inflammatory and antibacterial activities. It also has two major amino acid sequences with different modifications, including glycosylation, phosphorylation, and oxidation. To understand the potential biological role of CMP within the human body, there is a need to examine the extent to which CMP and CMP-derived fragments survive across the digestive tract, where they can exert these functions. In this study, three solid-phase extraction (SPE) methods—porous graphitized carbon (PGC), hydrophilic interaction liquid chromatography (HILIC), and C18 chromatography—were evaluated to determine which SPE sorbent is the most efficient to extract intact CMP and CMP-derived peptides from WPI and intestinal digestive samples prior to LC-MS/MS acquisition. The C18 SPE sorbent was the most efficient in extracting intact CMP and CMP-derived peptides from WPI, whereas the PGC SPE sorbent was the most efficient in extracting CMP-derived peptides from intestinal digesta samples.

## 1. Introduction

Kappa-casein-derived caseinomacropeptide (CMP) is a 64-amino-acid C-terminal fragment of bovine κ-casein released during cheesemaking via enzymatic hydrolysis by chymosin. CMP is the third most abundant protein component in cheese whey, making up 20% to 25% of the proteins by mass [1]. Sixty percent of CMP is glycosylated (referred to as glycomacropeptide (GMP)). Eleven glycan structures have been identified in bovine GMP [2], ten of which have an *O*-glycan core galactosyl-beta 1,3-N-acetylglucosamine, while the other has only N-acetylglucosamine. Five of these are found both in colostrum and mature milk, whereas the other six are only found in colostrum. The glycans on GMP are linked to the oxygen atoms of threonine (Thr) and serine (Ser) in the amino acid chain (*O*-linked glycans). These O-linked glycans present on GMP are composed of galactose (Gal), N-acetyl galactosamine (GalNAc), and N-acetyl neuraminic acid (NeuAc, or sialic acid) [3].

GMP is commercially available, and its current main use is for the nutritional management of phenylketonuria. Because its amino acid sequence does not contain phenylalanine, it is an excellent protein source for people who have phenylketonuria—an inherited disorder that prevents metabolism of phenylalanine [4].

CMP also has numerous bioactivities in vitro, including the ability to bind cholera toxin [5] and *E. coli* enterotoxin [6], inhibit bacterial and viral pathogens’ adhesion to Caco-2 cells [7,8], promote beneficial bifidobacterial growth [9], reduce intestinal epithelial cell barrier dysfunction [10], and modulate the immune system response [11,12,13]. To understand the potential biological role of digested CMP within the human body, there is a need to examine the extent to which CMP and CMP-derived fragments survive across the digestive tract, where they can exert these functions.

Our previous study identified the intact CMP in commercial CMP powders and a CMP standard through a top-down approach based on liquid chromatography and tandem mass spectrometry (LC-MS/MS) analysis without any clean-up procedure [14]. In our follow-up study, we obtained intestinal samples from participants fed CMP via nasojejunal tubes [15]. Unlike analysis of the highly purified commercial CMP powder, identifying the intact and digested CMP in intestinal digestive samples required extraction and purification prior to LC-MS/MS acquisition [15]. Intestinal digestive samples contain digested feeding material and a complex array of intestinal fluid components, including hormones, digestive enzymes, bile salts, mucus, and sloughed mucosal cells [16]. LC-MS-based analysis of CMP and CMP-derived peptides would be impaired without extraction and purification, as the complex intestinal matrix can interfere with analytical column binding and elution, and the presence of non-target molecules can inhibit the ionization and detection of target molecules. In the previous work [15], we applied ethanol precipitation and C18 solid-phase extraction (SPE) to isolate CMP and CMP-derived peptides from the intestinal samples. In the present work, we examine multiple SPE sorbents to determine which is optimal for analyzing these peptides in digestive fluids for future studies.

In SPE, a sample is dissolved in a mobile phase and passed over a solid-phase sorbent with specific physicochemical properties, ideally allowing differential interaction with analytes and non-target molecules to enable their separation prior to analysis. SPE sorbents commonly used to enrich and purify glycopeptides include C18, porous graphitized carbon (PGC), and hydrophilic interaction liquid chromatography (HILIC). The C18-based glycopeptide enrichment method is based on hydrophobic interaction between the relatively non-polar peptide moiety and the C18 chains of the stationary phase [15]. C18 has been used for lab-scale isolation of glycopeptides but has not been used commercially for GMP purification in the dairy industry. PGC retains glycopeptides through polar interactions between the glycan moiety and the graphite solid phase, along with planar–planar interactions between the glycan moiety and the graphite surface [17]. PGC has been used for oligosaccharide separations in dairy products [18], but not for GMP. HILIC has been commonly used to enrich intact N- and O-linked glycopeptides, which are retained in the polar stationary phase by dipole–dipole, ionic, and hydrogen interactions [19,20]. Though used at the lab scale for GMP isolation previously, HILIC-based enrichment may be inefficient for GMP and GMP-derived peptides because the O-linked glycopeptides’ structures are relatively small compared with the long peptide chain (an intact GMP has 64 amino acids, and the largest known O-glycan is a tetrasaccharide).

No previous study has examined which SPE method is most efficient to extract CMP and CMP-derived peptides from intestinal digestive samples. Therefore, in this study, we systematically compared C18, PGC, and HILIC to determine which SPE sorbent most efficiently extracts intact CMP and CMP-derived peptides from whey protein isolate (WPI) and intestinal digestive samples.

## 2. Materials and Methods

A commercial WPI and intestinal digestive sample obtained from one subject after consuming WPI were extracted using three SPE sorbents (C18, PGC, and HILIC) to determine which was most efficient for extracting intact CMP and CMP-derived peptides prior to LC-MS/MS analysis.

### 2.1. Intestinal Sample Collection

Intestinal samples were collected from one subject (male, 34 year old, BMI 22.3) by staff at the Good Samaritan Hospital (Corvallis, OR, USA). The subject was screened by the medical staff at the Good Samaritan Hospital Corvallis for study eligibility. The eligibility criteria were as follows: the absence of lactose and dairy protein intolerance, the absence of gastrointestinal disease, the absence of gastrointestinal surgery and nasal injury, and the absence of esophageal anomaly. The protocol was approved by the Good Samaritan Hospital Corvallis and Oregon State University Institutional Review Boards (IRB number: IRB 18-088; Corvallis, Oregon), and written consent was obtained from the subject. The subject was required to have a dairy-free diet for three days before the collection. Additionally, the subject fasted overnight before the fourth day. On the fourth day, the subject came to Good Samaritan Regional Medical Center for sample collection. A 140 cm, 10 Fr nasojejunal tube (CORTRAK* 2 Nasogastric/Nasointestinal Feeding Tube with Electromagnetic Transmitting Stylet, 20-9551TRAK2, Avanos Medical, Alpharetta, GA, USA) was inserted into the subject, and the position was checked to ensure that the tip had migrated to the proximal jejunum using the CORTRAK 2 Enteral Access System.

A 1 L protein shake containing 1282 kcal and made up of 68 g of WPI (Provon 290, Glanbia Nutritionals, Twin Falls, ID, USA), 140 g of sucrose, and 150 mL of heavy whipping cream (50 g fat) in water was consumed by the subject within 1 h. The subject also drank an additional 2 L of water during this period. Jejunal fluids were collected up to 3 h after protein shake consumption from the nasojejunal tube by gravity flow. After collection, the nasojejunal tube was removed. The collected intestinal samples were placed on dry ice and stored at −80 °C until analysis. The intestinal samples were thawed on ice and pooled together across the 3 h of digestive samples collected to study the extraction of CMP and CMP-derived peptides.

### 2.2. Solid-Phase Extraction of WPI and Intestinal Samples

C18 (Discovery^®^ DSC-18 SPE Tube, 500 mg, 6 mL, Sigma-Aldrich Inc., St. Louis, MO, USA) cartridges were rinsed with 5 mL of 18.2 MΩ water, 5 mL of 80% acetonitrile (ACN) with 0.1% trifluoroacetic acid (TFA), and 5 mL of water. Then, 100 µL of WPI (1 mg/mL in 18.2 MΩ water) or 25 μL of intestinal sample was loaded on the C18 column. These volumes were selected to load SPE columns with ~0.1 mg of protein (calculated for the intestinal sample based on the initial concentration of the WPI isolate feed and dilution factors for typical digestive secretions from the literature [21], as well as the known additional water intake by the participant (2 L)). Loaded samples in the C18 cartridge were washed with 6 mL of water and eluted with 5 mL of 80% ACN with 0.1% TFA.

PGC (Supelclean™ ENVI-Carb™ SPE Tube, 500 mg, 6 mL, Sigma-Aldrich Inc., St. Louis, MO, USA) cartridges were rinsed with 5 mL of 18.2 MΩ water, 5 mL of 80% ACN with 0.1% TFA, and 5 mL of water. Then, 100 µL of WPI (1 mg/mL in water) or 25 μL of intestinal sample was loaded on the PGC cartridge. The loaded samples in the PGC cartridge were washed with 6 mL of water and eluted with 5 mL of 80% ACN with 0.1% TFA.

HILIC (Hilicon^®^ iSPE-HILIC, 3 mL, 200 mg, 50 μm, 60 Å, HILICON, Umeå, Sweden) cartridges were sequentially rinsed with 3 mL of 95% ACN with 1% TFA, 3 mL of 18.2 MΩ water with 1% TFA, and 3 mL of 95% ACN with 1% TFA. Then, 100 μL of WPI (1 mg/mL in 95% ACN with 1% TFA) or 25 μL of intestinal sample was loaded on the HILIC cartridge. The loaded samples in the HILIC cartridge were washed with 4 mL of 95% ACN with 1% TFA and eluted with 3 mL of water with 1% TFA.

Both the washing solution and the elution solution in each SPE were collected, freeze-dried, and reconstituted in 200 μL of water for mass spectrometry analysis.

### 2.3. Mass Spectrometry Analysis

Samples were analyzed using a Waters nanoACQUITY UPLC (Waters, Milford, MA, USA) with an Orbitrap Fusion™ Lumos™ Tribrid™ mass spectrometer (Thermo Scientific, Waltham, MA, USA) in MS and MS/MS mode, as described previously [14], with the following modifications: The gradient elution using solvent A (100% 18.2 MΩ water with 0.1% formic acid) and solvent B (100% ACN with 0.1% formic acid) was modified from a 30 to a 60 min gradient, as follows: 0–10 min, 3–13% B; 10–32 min, 13–20% B; 32–36 min, 20–30% B; 36–44.5 min, 35–95% B; 44.5–54.5 min, 95–95% B; 54.5–55 min, 95–3% B; 55–60 min, 3–3% B. The MS/MS spectral acquisition *m*/*z* range was changed to 400–2000.

### 2.4. Data Analysis

Spectra were analyzed as described in our previous publication [14] by database searching in Byonic v.3.11.0 (Protein Metrics Inc., Cupertino, CA, USA ). We updated the potential O-linked glycosylations in the glycan library to include HexNAc alone on Ser/Thr for all searches.

The relative abundances of each CMP and CMP-derived peptide were calculated by the following equation: chromatogram peak area of individual (glyco)peptide ÷ chromatogram peak area of all (glyco)peptide × 100. All LC-MS/MS analyses were performed in technical duplicates, and the relative abundances of the peptides were averaged in the reported data. The counts of unique peptides were counts of peptides with differing amino acid sequences or PTMs.

### 2.5. Statistical Analysis

Single-factor analysis of variance followed by Tukey’s post hoc test was performed to compare summed peptide abundances and counts among the three SPE types for each sample type: WPI wash, WPI elution, intestinal sample wash, and intestinal sample elution. All statistical analyses were conducted using R software, version 7.2. Comparisons resulting in a *p*-value below 0.05 were considered evidence of a significant difference.

## 3. Results and Discussion

Enrichment and purification of glycopeptides are necessary prior to MS analysis to increase the sensitivity for glycopeptide detection and avoid ion suppression induced by the co-presence of non-target molecules in the digestive sample. To compare glycopeptide enrichment strategies, both WPI solutions and digestive samples were enriched and purified using three different types of SPE cartridges—C18, PGC, and HILIC. These enrichment techniques have been widely used for enriching glycopeptides from samples [15,17]. Counts and total abundances of identified CMP and CMP-derived peptides were compared to determine the most effective method to enrich CMP and CMP-derived peptides in WPI and intestinal samples. Both the elution and wash fractions from the SPE were collected from each column and analyzed to determine which method allowed the highest recovery of the analyte in the elution and the lowest loss of the analyte in the wash fraction.

### 3.1. CMP and CMP-Derived Peptides Found in Whey Protein Isolate

Across all six analyzed samples (C18-elution, PGC-elution, HILIC-elution, C18-wash, PGC-wash, and HILIC-wash), a total of 54 unique CMP and CMP-derived peptides were identified.

**Figure 1:**
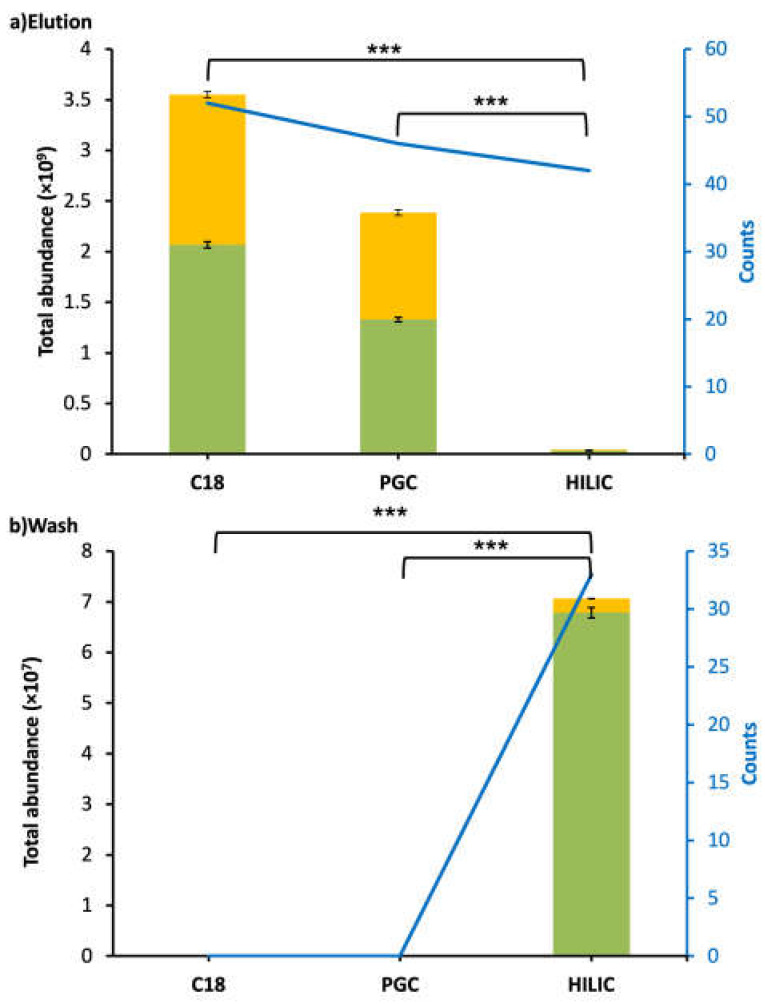
Bar graphs and line charts (blue) of total abundance and counts of CMP and CMP-derived peptides found in WPI after C18, PGC, and HILIC SPE. Abundances were based on the total peak abundances of all of the CMP and CMP-derived peptides. (**a**) Elution fraction. (**b**) Wash fraction. Peptides with O-glycan(s): yellow; peptides without O-glycan(s): green. Asterisks indicate statistically significant differences; *** means *p* ≤ 0.001.

**Figure 2:**
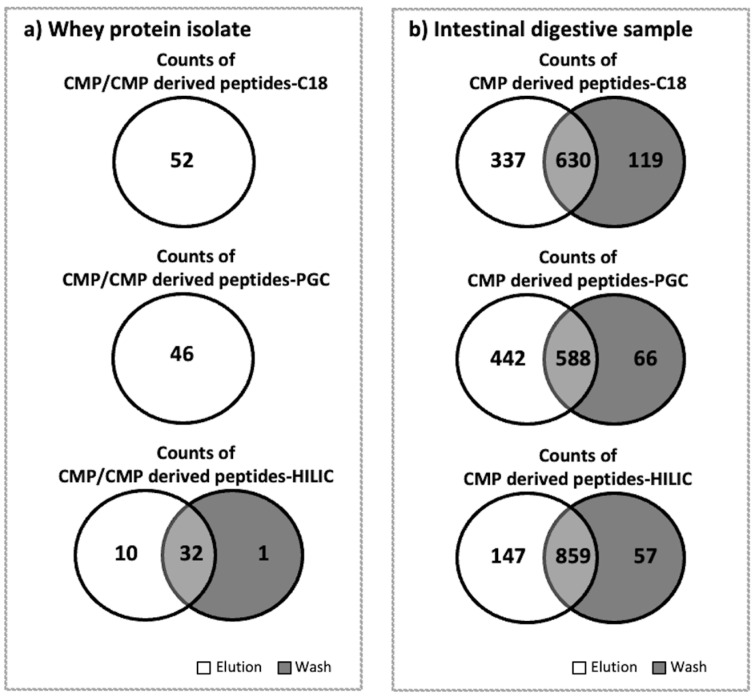
Venn diagrams for counts of CMP and/or CMP-derived peptides found in the elution and wash fractions of whey protein isolates or the intestinal digestive samples after the C18, PGC, and HILIC SPE. (**a**) Whey protein isolate. (**b**) Intestinal digestive sample. As no CMP or CMP-derived peptides were found in the C18 and PGC wash fractions in the whey protein isolate samples, a single circle is shown.

C18 was more effective for enriching CMP and CMP-derived peptides in WPI samples than PGC and HILIC. C18-elution had the highest counts (52) and abundance (3.55 × 10^9^), and no CMP or CMP-derived peptides were found in the C18-wash for WPI. The total abundance of peptides in the C18-elution was significantly higher than that in the PGC-elution and HILIC-elution (Tukey’s HSD, *p* < 0.001; Figure 1). PGC was the second-best-performing SPE for WPI, as the PGC-elution had the second-highest counts (46) and abundance (2.39 × 10^9^). No CMP or CMP-derived peptides were found in the PGC-wash. The total abundance of peptides in the PGC-elution was significantly higher than that in the HILIC-elution (Tukey’s HSD, *p* < 0.001). HILIC was inefficient for enriching CMP and CMP-derived peptides from WPI samples. The total abundances of peptides in both the elution (4.22 × 10^7^) and wash (7.06 × 10^12^) fractions of HILIC were extremely low. It is possible that CMP and CMP-derived peptides were trapped in the HILIC SPE column and could not be eluted by either the wash or elution fluids. Compared with C18 and PGC, which had no CMP or CMP-derived peptides present in the wash fraction, HILIC had large counts of CMP and CMP-derived peptides (32) that were present in both the elution and wash fractions (Figure 2a). Therefore, the separations of CMP and CMP-derived peptides on HILIC were not efficient. In all of the washes and elutions of WPI samples, 55.7–95.5% of CMP and CMP-derived peptides were found with O-glycosylation. The percentages of CMP with glycosylation for C18 (58.2%) and PGC (55.7%) were similar to those found in previous studies [22], indicating that around 60% of CMP had O-glycosylation (Figure 1).

### 3.2. CMP and CMP-Derived Peptides Found in the Intestinal Samples

**Figure 3:**
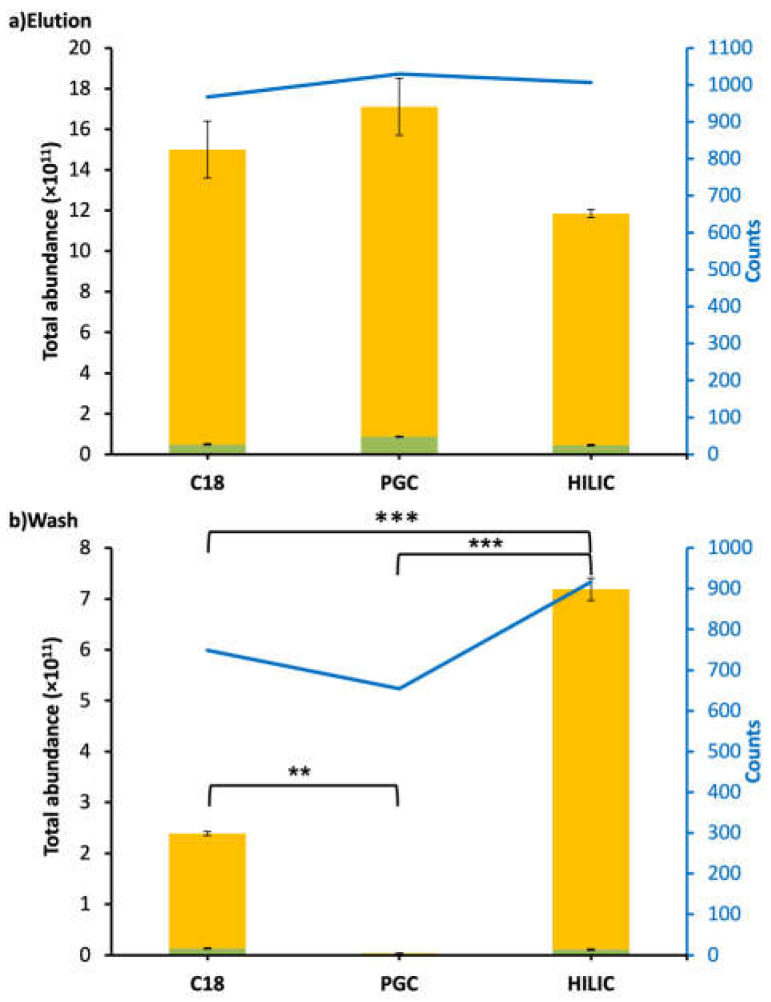
Bar graphs and line charts (blue) of total abundance and counts of CMP-derived peptides found in the intestinal digestive sample followed by C18, PGC, and HILIC SPE. Abundances were based on the total peak abundances of all of the CMP-derived peptides. (**a**) Elution fraction. (**b**) Wash fraction. Peptides with O-glycan(s): green; peptides without O-glycan(s): yellow. Asterisks indicate statistically significant differences; ** means *p* ≤ 0.01, *** means *p* ≤ 0.001.

Compared to the peptides found in WPI samples, a very low percentage (<0.07%) of intact CMP was found in intestinal samples. However, high counts and abundances of CMP-derived peptides were found in the intestinal samples. Across all six analyzed samples from the intestinal digesta (C18-elution, PGC-elution, HILIC-elution, C18-wash, PGC-wash, and HILIC-wash), a total of 1159 CMP and CMP-derived peptides were identified. PGC-SPE was the best-performing SPE for enriching CMP and CMP-derived peptides from intestinal samples compared with C18 and HILIC. The PGC-SPE elution had the highest counts (1030) and abundance (1.71 × 10^12^), and very few of these peptides were found in the PGC wash for the intestinal sample (Figure 3). The total abundance of peptides in the PGC-SPE elution was not statistically higher than that of C18 (1.49 × 10^12^) or HILIC (1.18 × 10^12^) (Figure 3). Five-hundred and eighty-eight CMP peptides overlapped in both the elution (of 1030 total peptides) and wash (of 654 total peptides) of PGC-SPE (Figure 2b). This ratio of overlapped to total in the elution (588:1030) was low compared with C18 (630:967) and HILIC (859:1006) (Figure 2b). PGC-SPE performed well for enriching the CMP-derived peptides in the intestinal sample. Though C18-SPE was the most effective sorbent for enriching CMP and CMP-derived peptides in WPI, it was the second-best-performing sorbent for enriching CMP-derived peptides in the intestinal sample. The C18 elution had the third-highest counts of CMP-derived peptides (n = 967), whereas 630 peptides were found to be overlapped in both the elution (n = 967) and wash (n = 749) of C18 SPE. HILIC was not efficient for enriching CMP-derived peptides from intestinal samples. The abundances of CMP and CMP-derived peptides in the HILIC elution were not significantly lower than those in the C18 or PGC elutions, but a large amount of CMP-derived peptides was detected in the wash. The high abundance of overlapping peptides found in the wash and elution fractions from the HILIC column may represent peptides that are retained weakly by the HILIC sorbent due to relatively low polarity. A limitation of this study is that a pooled intestinal sample across 3 h of digestion from a single subject was examined, rather than a larger number of samples. It is possible that differences in each person’s digestive capacity could produce a different profile of CMP-derived peptides, which could lead to alterations in the observed effectiveness of extraction of these peptides across the C18, PGC, and HILIC columns.

We found that the site distribution of CMP-derived peptides within the overall CMP sequence was similar for the PGC, C18, and HILIC SPE elutions (Figure S1).

### 3.3. Comparison of CMP and CMP-Derived Peptides Found in Whey Protein Isolate and the Intestinal Samples in C18 and PGC Elutions

**Figure 4:**
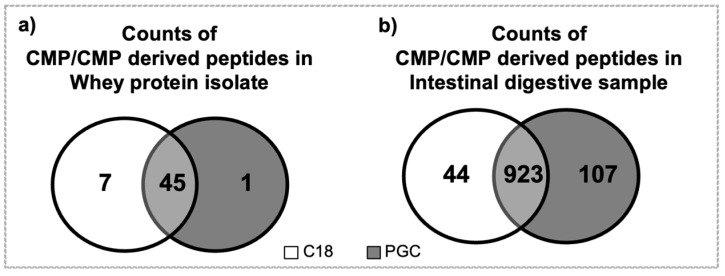
Venn diagrams for counts of CMP and CMP-derived peptides found in the C18 (white) and PGC (grey) elution fractions of (**a**) whey protein isolate and (**b**) the intestinal digestive samples.

Forty-five CMP and CMP-derived peptides were found to overlap in both elutions of C18 (of 52 total peptides identified) and of PGC-SPE (of 46 total peptides identified) of whey protein isolate (Figure 4). The relative abundance of the seven CMP-derived peptides unique to the C18 elution in WPI was 0.12%, and the relative abundance of the one CMP peptide unique to the PGC elution in WPI was 0.03%. Nine-hundred and twenty-three CMP and CMP-derived peptides were found to overlap in both elutions of C18 (of 967 total peptides) and in PGC-SPE (of 1030 total peptides) of the intestinal digestive samples. For the intestinal digestive samples, the relative abundance of the 44 CMP-derived peptides unique to the C18 elution was 0.03%, and the relative abundance of the 107 CMP peptides unique to the PGC elution was 0.14%. These results also suggest that the C18 SPE is more effective than PGC in WPI, and that PGC is more effective for intestinal digestive samples but, in general, both approaches were effective for both sample types, as the relative abundance of peptides unique to either methodology was relatively low.

The C18-based glycopeptide enrichment method is based on hydrophobic interaction between the relatively non-polar peptide moiety and the C18 chains of the stationary phase [23]. PGC retains glycopeptides through polar interactions between the glycan moiety and the graphite solid phase, along with planar–planar interactions between the glycan moiety and the graphite surface [17].

**Figure 5:**
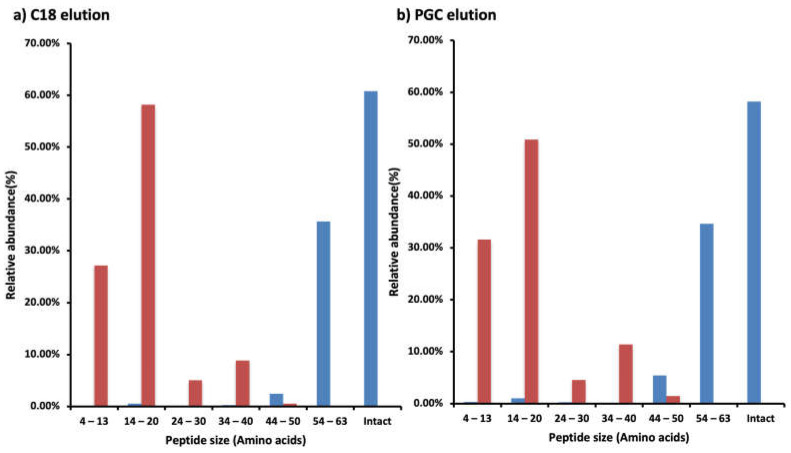
Bar graphs of the relative abundance of CMP and CMP-derived peptides based on peptide size (based on counts of amino acids) found in the WPI (blue) or the intestinal digestive sample (red) in the (**a**) C18 SPE or (**b**) PGC elution fractions. Relative abundances were calculated from the sum of peak abundances of the CMP and/or CMP-derived peptides in the labeled range of peptide size divided by the total peak abundances of the CMP and/or CMP-derived peptides in the WPI or the intestinal digestive sample in the fraction.

Though C18 was the most effective SPE for WPI, PGC performed somewhat better for the intestinal digestive sample. A key factor that could be responsible for this variation is the decreased lengths of the peptide moieties present in the intestinal sample compared with the WPI feed. In the WPI C18-SPE elution fraction, most peptides were intact CMP with 64-amino-acid peptide moieties, whereas in the intestinal sample most peptides ranged from 4 to 20 amino acids (Figure 5a). The same trend of the relative abundance of peptide size was found in the PGC-SPE elution (Figure 5b). The minimum length of the peptides was four amino acids in the intestinal digestive sample; shorter peptides were likely present as well, but our data analysis using Proteome Discoverer did not allow for identification of shorter peptides. There were eight forms (0.07% relative abundances) of intact CMP in the intestinal digestive sample in the C18 elution and six forms (0.04% relative abundances) in the PGC-SPE elution. All of the intact CMP present in the intestinal digestive sample was also present in the elution in WPI. Almost all of the CMP was partially digested by proteases in the human GI tract.

The proportion of the total measured relative abundance of CMP and CMP-derived peptides that had glycosylation was lower in the intestinal sample than in the WPI sample (Figure 1 and Figure 3). Digestion released many non-glycosylated CMP-derived peptides from both ends of the overall molecule. These non-glycosylated peptides represent a larger fraction of the total measured abundance of CMP and CMP-derived peptides, likely due to their suppression of glycopeptide ionization, as glycopeptides have a much poorer ionization efficiency than peptides [24]. Notably, the human digestive tract does not secrete enzymes with the capacity to degrade the O-glycan moiety of the CMP, so any O-glycans initially present in the WPI were likely still present in the intestinal samples.

## 4. Conclusions

This paper is the first to examine which SPE method is most efficient to extract CMP and CMP-derived peptides from WPI and intestinal digestive samples. Most of the CMP in the commercial dairy protein was in the intact form. In the digestive samples, almost all of the intact CMP was digested, and the CMP-derived peptides mostly ranged from 4 to 20 amino acids in length. C18-SPE was the best SPE for enriching CMP and CMP-derived peptides from WPI. Therefore, C18-SPE should be used for samples containing mostly intact CMP. PGC-SPE performed somewhat better than C18 for enriching the CMP-derived peptides present in the intestinal digestive samples. Therefore, PGC-SPE is recommended for samples containing mostly smaller CMP-derived peptides. As CMP mostly did not survive intact but, rather, was present as CMP-derived peptides in the upper intestine, future research should investigate the bioactivities of these CMP-derived peptides. These partially digested CMPs are more likely than intact CMPs to exert biological actions in the intestine.

## Data Availability

The data presented in this study are available in the article and the Supplementary Materials.

## Supplementary Materials

The following supporting information can be downloaded at: https://www.mdpi.com/article/10.3390/foods12020299/s1, Figure S1: Site-specific distribution of CMP-derived peptides across the CMP sequence found in the intestinal samples in C18-elution (red), PGC-elution (blue), and HILIC-elution (green). Table S1: The CMP and CMP-derived peptides found in the WPI in C18-elution, PGC-elution, HILIC-elution, C18-wash, PGC-wash, and HILIC-wash, with peptide sequence, number of phosphorylations, number of oxidations, number of O-glycosylations, and calculated mass. Table S2: The CMP and CMP-derived peptides found in the intestinal samples in C18-elution, PGC-elution, HILIC-elution, C18-wash, PGC-wash, and HILIC-wash, with peptide sequence, number of phosphorylations, number of oxidations, number of O-glycosylations, and calculated mass.
