# Supplementary material for: Comparison of Solid-Phase Extraction Sorbents for Monitoring the In Vivo Intestinal Survival and Digestion of Kappa-Casein-Derived Caseinomacropeptide
Source: Foods. 2023 Jan 8;12(2):299. doi: 10.3390/foods12020299 (PMC9858392; doi:10.3390/foods12020299)
Supplement: Supplementary file 1 [file foods-12-00299-s001.zip › Paper 2 Sup Figures and tables.pdf]

Comparison of Solid-Phase Extraction Sorbents for Monitoring the In Vivo Intestinal Survival and Digestion of Kappa-Casein-Derived Caseinomacropeptide

Yunyao Qu [1,2], Bum-Jin Kim [2], Jeewon Koh [2] and David C. Dallas [2,*]

[1] Department of Food Science & Technology, Oregon State University, Corvallis, OR 97331, USA
[2] Nutrition Program, School of Biological and Population Health Sciences, College of Public Health and Human Sciences, Oregon State University, Corvallis, OR 97331, USA
* Correspondence: dave.dallas@oregonstate.edu

Table of Content

FIGURE S1. Site-specific distribution of CMP-derived peptides across the CMP sequence found in the intestinal samples in C18-elution (Red), PGC-elution (Blue) and HILIC-elution (Green)..S-2
TABLE S1. The CMP and CMP-derived peptides found in the WPI in C18-elution, PGC-elution, HILIC-elution, C18-wash, PGC-wash, HILIC-wash with peptide sequence, number of phosphorylation, number of oxidations, number of O-glycosylations and calculated mass...S-3
TABLE S2. The CMP and CMP-derived peptides found in the intestinal samples in C18-elution, PGC-elution, HILIC-elution, C18-wash, PGC-wash, HILIC-wash with peptide sequence, number of phosphorylation, number of oxidations, number of O-glycosylations and calculated mass...S-3

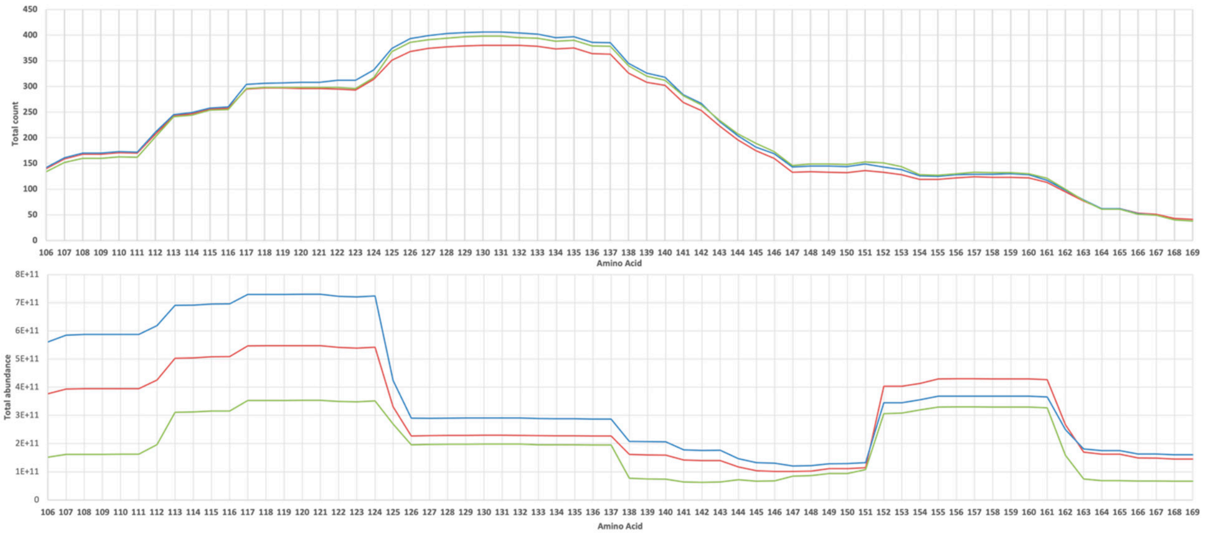

Figure S1. Site-specific distribution of CMP-derived peptides across the CMP sequence found in the intestinal samples in C18-elution(Red), PGC-elution(Blue) and HILIC-elution(Green).

Table S1. The CMP and CMP-derived peptides found in the WPI in C18-elution, PGC-elution, HILIC-elution, C18-wash, PGC-wash, HILIC-wash with peptide sequence, number of phosphorylation, number of oxidations, number of O-glycosylations and calculated mass.

*Excel S-1

Table S2. The CMP and CMP-derived peptides found in the intestinal samples in C18-elution, PGC-elution, HILIC-elution, C18-wash, PGC-wash, HILIC-wash with peptide sequence, number of phosphorylation, number of oxidations, number of O-glycosylations and calculated mass.

*Excel S-2
